# Personal Explanation

**Published:** 1896-05

**Authors:** 


### PERSONAL EXPLANATION.

Our attention has been called to the following paragraph taken from the letter of the New York correspondent of the *Pacific Stomatological Gazette* of March:

> "Much curiosity has arisen regarding the dismissal of Professor Truman from the secretaryship of the University of Pennsylvania. We were told by a Philadelphia professor that he was not given an opportunity to resign."

This, while in the main correct, will doubtless lead to wrong impressions injurious to all parties concerned.

The change in administration in the University brought with it a modification of former methods, which led indirectly to the retirement of Dr. Truman from the position of dean (not secretary) of the department of dentistry. This action had no relation to nor was it any reflection on his work past or present, nor did it affect his position as professor in the University.

